# Medication Adherence Among Patients of Type II Diabetes Mellitus and Its Associated Risk Factors: A Cross-Sectional Study in a Tertiary Care Hospital of Eastern India

**DOI:** 10.7759/cureus.33074

**Published:** 2022-12-29

**Authors:** Jyotiranjan Sahoo, Sambedana Mohanty, Arijit Kundu, Venkatarao Epari

**Affiliations:** 1 Community Medicine, Siksha 'O' Anusandhan Deemed to be University Institute of Medical Sciences and SUM Hospital, Bhubaneswar, IND; 2 Community Medicine, Shri Ramkrishna Institute of Medical Science and Sanaka Hospitals, Bhubaneswar, IND

**Keywords:** health related quality of life, morisky medication adherence scale-8, health correlates, risk score, patient compliance

## Abstract

Background: Type 2 diabetes mellitus is a major public health problem. Adherence to anti-diabetic medications improves glycaemic control, which in turn prevents complications as well as reduces out-of-pocket expenditure. The World Health Organization highlights that the impact of interventions directed to improve adherence has far greater implications than specific medical interventions. There are several factors that contribute to poor adherence. Not many studies have been conducted to explore adherence to diabetes medications in eastern India.

Objectives: To measure medication adherence among patients suffering from diabetes. To determine the various risk factors influencing adherence to medication. To find out the association of health-related quality of life with adherence to medication.

Methodology: A hospital-based cross-sectional study was conducted in the outpatient Department of General Medicine and Endocrinology of a tertiary care hospital in eastern India from January to March 2020. Adult subjects, who were diagnosed with type 2 diabetes mellitus for at least six months, were interviewed using a pretested, structured questionnaire containing 8-item Morisky Medication Adherence Scale (MMAS-8) to determine adherence to diabetic medications. Data were analysed in SPSS version 27 (IBM Corp., Armonk, NY, USA).

Results: The mean age of the 331 participants interviewed was 53.40 (SD 11.0) years and the majority were males (57.1%). Medication adherence of 34.14% (n=113) was found among the subjects. Having any comorbidity, positive family history of diabetes and the habit of current alcohol intake increased the odds of poor adherence by 3.26 times, 1.88 times, and 2.35 times respectively in binary logistic regression analysis. Those following a diabetic diet had a protective effect, decreasing poor medication adherence by 79.6%. Poor medication adherence increased by 1.077 times with every one-day increase in unhealthy days.

Conclusion: The medication adherence was 34.14% and as compared to other similar studies medication adherence in the study population was poor and was associated with unhealthy days.

## Introduction

Medication adherence is defined as "the degree to which the person’s behaviour corresponds with the agreed recommendations from a health care provider" [1]. In simple terms, it can be explained as an active, voluntary patient involvement to adopt an acceptable behaviour to produce desirable therapeutic results [2,3]. Lack of adherence to medication results in unfavourable outcomes and higher financial burdens [2]. Despite causing an estimated 125 thousand avoidable deaths every year and preventable healthcare costs of $100 billion annually, non-adherence to medication is mostly ignored by practising physicians [4]. In 2003, the World Health Organization highlighted that the impact of interventions directed to improve adherence has far greater implications than specific medical interventions [5].

Globally, while the burden due to chronic diseases is increasing, there is no significant improvement in adherence to treatment [6] as reflected by the medication adherence rates of about 50% to 60% for hypertension or diabetes despite good insurance coverage [4]. In developed countries, just over 50% of patients adhere to the prescribed medications, while it is still lower in developing countries [6,7].

Diabetes is one of the commonest chronic diseases in the world with rising prevalence [3]. It is becoming a major public health challenge affecting more than 425 million people worldwide [7,8]. There are about 62.4 million people with type 2 diabetes and about 77 million with pre-diabetes in India as per the INdiaDIAbetes report, which is likely to rise to 101 million by the year 2030 [3]. Thus, India is considered the “diabetes capital of the world” [9] with a prevalence varying from 5.6% in rural areas to 12.1% in major cities [7]. Diabetes has also contributed the highest to regional mortality with 1,065,052 deaths due to diabetes in 2013 [7]. In 2016 alone, diabetes was the direct cause of 1.6 million deaths and in 2012, high blood glucose was the cause of another 2.2 million deaths [10].

Hyperglycaemia, or raised blood sugar, is an outcome of uncontrolled diabetes [10] that results in several micro (retinopathy, nephropathy) and macrovascular (coronary artery disease) complications [11,12]. Good metabolic control can delay the onset and progression of complications in both type 1 and 2 diabetes [8,11]. Thus, diabetics require lifelong treatment with medications and follow-up. Adherence to anti-diabetic medications improves glycaemic control, which in turn prevents complications and has a better prognosis. Further, it is cost-effective as it reduces the frequency of hospitalization and cost associated with complications [11]. Self-care in the form of adherence to diet and drugs, blood glucose monitoring, foot care, exercise and recognition of the symptoms, are crucial elements that are needed for secondary prevention [5,7,9].

Several factors contribute to non-adherence, which include out-of-pocket expenditure, literacy, lack of awareness, and inadequate family or community support. Multiple diseases and polypharmacy among older adults are further challenges to medication adherence. Unequal distribution of health providers between urban and rural areas and cultural norms are barriers to compliance with medications. Forgetfulness because of mental comorbidity too contributes to non-adherence. Cochrane Review concluded that one explanation for non-effectiveness is the lack of a thorough understanding of the adherence problems [5-7,9,11,12].

Yet, unlike better-known causes of death such as heart attack or cancer, the effects of medication non-adherence are usually invisible to patients, their families, and the medical profession [4]. Not many studies have been conducted to explore adherence to diabetes medications in eastern India. Thus, this study was undertaken with the following objectives: to measure medication adherence among patients having type II diabetes; to determine the various risk factors affecting adherence to medication in the study population; and to find out the association of health-related quality of life (HRQOL) with adherence to medication in the study population.

## Materials and methods

Study setting

A hospital-based cross-sectional study was conducted in the Department of General Medicine and Endocrinology of the Institute of Medical Sciences and SUM Hospital, Bhubaneswar, Odisha, India from January to March 2020. Adult patients (18 years or above) who were diagnosed with type 2 diabetes mellitus for at least six months before the study were recruited from the outpatient department.

Sample size

Based on a recent facility-based cross-sectional study [13] in the Indian population, which reported a non-adherence of 32.7% (95% CI 27.2 - 38.6), using OpenEpi software version 3.01, with an absolute precision of 5.5%, power of 80%, at 95% confidence the sample size was calculated to be 280. Bearing in mind a non-response rate of 20%, the final sample of 328 was considered adequate. 

Sampling method

Every alternate patient attending the clinic who satisfied the eligibility criteria was selected. Those patients who were severely ill/non-ambulatory, had problems with communication, with psychiatric illness (physician-diagnosed illness reported by the participant) or of more than 80 years (likelihood of more recall bias) were excluded from the study. The study procedure was explained to every patient and written consent was obtained before enrollment into the study.

Questionnaire

An interviewer-administered structured questionnaire in the English language was developed using the 8-item Morisky Medication Adherence Scale (MMAS-8) (a validated instrument [14,15], available freely in the public domain) to determine adherence to diabetic medications. It was pretested on 33 subjects (10% of the total sample size) and necessary modifications were incorporated before administration. The questionnaire had four sections. Section one contained socio-demographic factors (age, gender, religion, caste, education, occupation [unemployed/gainfully employed], marital status, residence [urban/rural], family size and monthly family income). Students and females who were housewives were included under unemployed. Section two captured medical history related to diabetes, current co-morbid conditions, and personal history (smoking/alcohol). The third section pertained to medication adherence, MMAS-8. In this scale, the first seven questions had binary response categories (yes/no) while the eighth item had a five-point Likert response. Previous studies [15] suggested that MMAS-8 had better psychometric properties, with sensitivity and specificity of 93% and 53% respectively and Cronbach's alpha value of 0.83. The fourth section captured information related to the health-related quality of life. We used the Healthy Days Core Module developed by the Centers for Disease Control and Prevention (CDC HRQOL-4, a validated instrument) to determine health-related quality of life. This module had four questions; the first question was self-rated general health on a five-point Likert scale from excellent to poor, the next two questions were used to calculate unhealthy days and the last question was used to calculate days of activity limitation in the last 30 days. The reliability of this scale was high at 0.75 for self-reported health and healthy days. 

Data analysis

All the data were analysed in SPSS version 27 (IBM Corp., Armonk, NY, USA). The categorical variables were expressed in numbers and percentages and the association between the two groups was calculated using Chi-square/Fischer exact test. The quantitative variables were expressed as mean (standard deviation) or median (interquartile range). Shapiro-Wilk test was used to determine the normality of data and accordingly either the independent samples t-test or Mann-Whitney U test was used to compare the means between the two groups. We also conducted a binary logistic regression analysis to eliminate confounders in predicting non-adherence to the medication. All the variables which were found to be significant (P-value <0.05) in univariate analysis were included in the multivariate logistic regression model. Unadjusted and adjusted odds ratios with a 95% confidence interval (CI) were reported. A p-value of less than 0.05 was considered statistically significant.

## Results

This study tried to find out the association between different identified risk factors and adherence to diabetic medication. A total of 352 patients were approached and out of them 12 subjects did not give consent, two were excluded based on exclusion criteria and seven had missing data during the data cleaning process. Thus, the final sample size was 331 (non-response rate=6.34%). The mean age of the participants was 53.40 (SD 11.0) years and the majority were males (57.1%). The socio-demographic characteristics are described in Table 1. As per MMAS-8, we found an appropriate medication adherence of 34.14% (n=113) among the subjects. In univariate analysis, none of the socio-demographic variables was associated with medication adherence at a significant level (Table 1).

**Table 1:** Association of socio-demographic factors and adherence to medication in the study population (N = 331) IQR=Interquartile range, *Mann-Whitney U test, # Fischer exact test and for the rest of the variables Chi-squared test was applied.

Variables	Poor-adherence to medication	Good adherence to medication	Total	p-value
Age median (IQR)*	55.0 (46.0-62.0)	52.0 (45.5-60.0)	54.0 (46.0-60.0)	0.307
Gender n (%)				
Male	125 (57.3)	64 (56.6)	189 (57.1)	0.903
Female	93 (42.7)	49 (434)	142 (42.9)	
Residence n (%)				
Urban	151 (69.3)	88 (77.9)	239 (72.2)	0.097
Rural	67 (30.7)	25 (22.1)	92 (27.8)	
Occupation n (%)				
Unemployed	102 (46.8)	49 (43.4)	151 (45.6)	0.553
Gainfully employed	116 (53.2)	64 (56.6)	180 (54.4)	
Religion n (%)^ #^				
Hindu	208 (95.4)	109 (96.5)	317 (95.8)	0.779
Others	10 (4.6)	4 (3.5)	14 (4.2)	
Education n (%)				
Up to high school	49 (22.5)	14 (12.4)	63 (19.0)	0.085
Intermediate	129 (59.2)	76 (67.3)	205 (61.9)	
Graduate and above	40 (18.3)	23 (20.4)	63 (19.0)	
Marital status^# ^n(%)				
Married	209 (95.9)	111 (98.2)	320 (96.7)	0.343
Un-married	9 (4.1)	2 (1.8)	11 (3.3)	
Caste n (%)				
General	171 (78.4)	89 (78.8)	260 (78.5)	0.946
Others	47 (21.6)	24 (21.2)	71 (21.5)	
Family size n (%)				
≤ 5	147 (67.4)	75 (66.4)	222 (67.1)	0.846
> 5	71 (32.6)	38 (33.6)	109 (32.9)	
Monthly income in thousand INR (Indian National Rupee) median (IQR)*	29.4 (25.7-29.4)	29.4 (24.0-29.6)	29.4 (24.0-29.6)	0.261

Comorbidities like hypertension (45.6%), arthritis (9.7%), asthma/other chronic respiratory diseases (7.9%), chronic kidney disease (2.1%), and cardiovascular diseases (0.6%) were noted among the study participants. In univariate analysis, having any comorbidity, especially hypertension, or having one or more comorbidities significantly increased the odds of poor medication adherence. Subjects with the habit of smoking or alcohol intake, having a positive family history of diabetes and having complications due to diabetes had increased odds of poor adherence. A higher proportion of participants having a positive lifestyle like following a diabetic diet (92.0%) and doing physical exercise (77.0%) had better medication adherence (Table 2).

**Table 2:** Association of medical history/personal habits and adherence to medication in the study population (N = 331) * Statistically significant

Variables	Adherence		Total	Odds Ratio
	Poor Good			(*significant)
	n (Row %)	n (Row%)	n (Column %)	(95% CI)
Hypertension				
No	99 (55.0)	81 (45.0)	180 (54.4)	1
Yes	119 (78.8)	32 (21.2)	151 (45.6)	3.04* (1.87 – 4.96)
Any comorbidity				
No	82 (51.9)	76 (48.1)	158 (47.7)	1
Yes	136 (78.6)	37 (21.4)	173 (52.3)	3.41* (2.11 – 5.50)
Co-morbidities				
None	82 (37.6)	76 (67.3)	158 (47.7)	1
One	110 (50.5)	29 (25.7)	139 (42.0)	3.52* (2.10 – 5.88)
Multiple	26 (11.8)	8 (7.1)	34 (10.3)	3.01*(1.28 – 7.06)
Family history of diabetes +ve				
No	100 (57.5)	74 (52.5)	174 (52.6)	1
Yes	118 (54.1)	39 (34.5)	157 (47.4)	2.24* (1.39 – 3.58)
Current smoker				
No	162 (62.1)	99 (37.9)	261 (78.9)	1
Yes	56 (25.7)	14 (12.4)	70 (21.1)	2.44* (1.29 – 4.62)
Current alcoholic				
No	175 (62.7)	104 (37.3)	279 (84.3)	1
Yes	43 (19.7)	9 (8.0)	52 (15.7)	2.84* (1.33 – 6.06)
On Diabetic diet				
Yes	154 (70.6)	104 (92.0)	258 (77.9)	1
No	64 (87.7)	9 (12.3)	73 (22.1)	4.80*(2.29 – 10.07)
Physical activity				
Yes	123 (56.4)	87 (77.0)	210 (63.4)	1
No	95 (78.5)	26 (21.5)	121 (36.6)	2.58* (1.55 – 4.32)
Complications				
No	149 (62.3)	90 (37.7)	239 (72.2)	1
Yes	69 (31.7)	23 (20.4)	92 (27.8)	1.81* (1.06 – 3.11)

The duration of diabetes (time since the first diagnosis) ranged from six to 240 months with a mean duration of 50.65 months (SD 43.59). The fasting blood sugar level was found to be significantly lower among subjects with good medication adherence compared to their counterparts. Although equal proportions of subjects who are taking oral hypoglycemic agents (OHA) had good and poor adherence those who are taking insulin had better adherence (p-value - 0.015). Other factors related to diabetes like duration of diabetes, laboratory parameters (post prandial blood sugar [PPBS] and glycosylated haemoglobin [HbA1c]), and various types of expenditure were not associated with medication adherence (Table 3).

**Table 3:** Association of investigation and treatment-related factors with adherence to medication in the study population *Mann-Whitney test was applied HbA1c=glycosylated haemoglobin

Variables	Poor adherence to medication (Mean ± SD)	Good adherence to medication (Mean ± SD)	Total (Mean ± SD)	p-value
Duration of diabetes in months*	53.10 ± 45.62	45.94 ± 39.12	50.65 ± 43.59	0.114
Fasting Blood Sugar* (n=197)	152.2 ± 39.7	144.4 ± 52.9	149.7 ± 43.79	0.003
Post Prandial Blood Sugar (n=166)	227.6 ± 64.9	227.3 ± 68.7	227.5 ± 65.95	0.976
HbA1c (n=64)	6.83 ± 1.55	6.88 ± 0.57	6.84 ± 1.34	0.884
Expenses on drugs	1163.8 ± 1074.4	1110.6 ± 934.8	1146.9 ± 1029.3	0.928
Expenses on transport	597.5 ± 692.9	514.5 ± 598.8	570.2 ± 662.6	0.263
Expenditure on Investigation	921.7 ± 1010.6	844.4 ± 823.4	895.6 ± 949.4	0.971
Expenditure on consultation	605.4 ± 503.1	547.7 ± 445.3	585.4 ± 482.8	0.217
Total expenditure* (Median ± IQR)	1900 ± 3000	2000 ± 1888	1950 ± 2600	0.444
Drug category n (%)				
Oral Hypoglycaemic Agents	165 (75.7)	85 (75.2)	250 (75.5)	0.015
Insulin	26 (11.9)	23 (20.4)	49 (14.8)	
Both	27 (12.4)	5 (4.4)	32 (9.7)	
Counselling n (%)				
Present	201 (92.2)	110 (97.3)	311 (94.0)	0.063
Absent	17 (7.8)	3 (2.7)	20 (6.0)	
Counselled by n (%)				
Physician	191 (95.0)	103 (93.6)	294 (94.5)	0.607
Others	10 (5.0)	7 (6.4)	17 (5.5)	
Number of consultations n (%)				
None	31 (14.2)	12 (10.6)	43 (13.0)	0.637
≤ 2	108 (49.5)	57 (50.4)	165 (49.8)	
> 2	79 (36.2)	44 (38.9)	123 (37.2)	

While measuring the quality of life of participants using the CDC HRQOL-4 questionnaire, we found that 247 (74.6%) subjects had good self-rated health. The mean duration of limitation of activity and unhealthy days was 3.57 (SD 5.34) and 7.77 (SD 7.76) respectively. Unhealthy days were significantly higher among patients with poor adherence (8.89 ± 8.44) (Table 4).

**Table 4:** Association of health-related quality of life with adherence to medication in the study population *Mann Whitney test applied IQR=interquartile range

Variables	Adherence Poor Good		Total (Mean ± SD)	P value
Self-rated health n (%)				
Poor	55 (25.2)	29 (25.7)	84 (25.4)	0.931
Good	163 (74.8)	84 (74.3)	247 (74.6)	
Days of activity limitation* (media ± IQR)	2 ± 4	2 ± 4	2 ± 4	0.132
Unhealthy days* (mean ± SD)	8.89 ± 8.44	5.63 ± 5.69	7.77 ± 7.76	0.001

In the binary logistic regression model, ‘having any comorbidity’ was included and ‘hypertension’ and ‘having multiple comorbid conditions’ were excluded as they were derivatives of the former. Also, since the response rate for the fasting blood sugar was close to 50%, we excluded this variable from the regression model. In the final analysis, we put forth 10 variables into the regression model using the stepwise forward likelihood method. Our model significantly predicted medication adherence (p-value <0.001), and had a good fit (Hosmer and Lemeshow test of goodness of fit, p-value = 0.170), with a Negelkerke's pseudo-R square of 0.281. Having any comorbidity, positive family history and habit of current alcohol drinking increased the odds of poor adherence by 3.26 (95% CI 1.93-5.50), 1.88 (95% CI 1.11-3.17), and 2.35 (95% CI 1.03-5.36) respectively. Following a diabetic diet decreased poor medication adherence by 79.6%. Poor medication adherence increased by 1.077 (95% CI 1.03-1.12) with every one-day increase in unhealthy days (Table 5).

**Table 5:** Binary logistic regression analysis for poor adherence to medication (forward conditional LR model) DM=diabetes mellitus, CI=confidence interval, OR=odds ratio, LR=likelihood ratio. The model was adjusted for smoking, physical activity, drug category, counselling and complication.

Variable	Unadjusted Odds ratio			Adjusted Odds ratio			
	Beta	OR	95% CI	Beta	OR	95% CI	
Any comorbidity							
Absent		1	-		1	-	
Present	1.226	3.407	2.11-5.50	1.182	3.260	1.93-5.50	
Family history							
Absent		1	-		1	-	
Present	0.806	2.239	1.39-3.58	0.634	1.885	1.11-3.17	
Alcohol habit							
Absent		1	-		1	-	
Present	1.044	2.839	1.33-6.06	0.857	2.357	1.03 -5.36	
Following DM diet							
No		1	-		1	-	
Yes	-1.569	0.208	0.09-0.43	-1.542	0.214	0.09-0.46	
Unhealthy days	0.068	1.070	1.03-1.11	0.075	1.077	1.03-1.12	

## Discussion

This study was conducted to measure adherence to medications among diabetic patients and explore the determining factors as well as to associate it with quality of life.

According to MMAS-8, good adherence was found only among 113 (34.14%) subjects in our setting. Many studies have been conducted in different parts of the country but adherence measured in all other settings was different owing to the tool used to measure the same. Those using the MMAS-8 have also reported varying rates of medication adherence as reported by Sankar et al. that found 74% of subjects had poor adherence [11], while Mukherjee et al. found 42.3% of subjects had poor adherence [9]. A study by Venkatesan et al. found 45.4% of subjects with poor adherence [5]. But, in contrast, Pattnaik et al. reported that only 9.7% of subjects had low compliance [8]. As per the study by Anurupa et al., 55% of participants were found to be having inadequate adherence to medications [7]. Arul Mozhi et al. in their study found that 50.7% of subjects were not having adequate compliance with medications [16]. Sharma et al. found that subjects had very low adherence i.e. 16.6% [17]. Differences in rates of medication adherence may be attributed to various study settings, sociodemographic variables and tools used.

Using the logistic regression model, it was found that the odds of poor adherence increased by 3.26 (95% CI 1.93-5.50) in presence of any comorbidity. A study by Venkatesan et al. found that chances of poor adherence were higher by 1.6 (1.04-2.5) times among those who have hypertension [5].

A positive family history and current alcohol drinking habit even escalated the chances of poor adherence by 1.88 (95% CI 1.11-3.17) and 2.35 (95% CI 1.03 -5.36) times respectively. Such results have not been interpreted in other studies. Following a diabetic diet decreased poor medication adherence by 79.6%. None of the previous studies has shown such an association. Poor medication adherence increased by 1.077 (95% CI 1.03-1.12) with every one-day increase in unhealthy days. These findings were unique to our study.

Limitations

As it is a hospital-based study, the results cannot be generalized. Being cross-sectional in nature the causal association cannot be established. Further studies can be done to evaluate the effect of adherence on glycemic control and reduction in the onset or progress of complications.

## Conclusions

In our study, we found that adherence to medications for diabetes was poor as compared to studies conducted in other similar settings. The factors associated with poor adherence were the presence of any comorbidity, a positive family history of non-communicable diseases and current alcohol consumption, while following a diabetic diet was associated with better medication adherence. Health education and lifestyle modifications may improve medication adherence as well as it will delay the progression of the disease.
